# Functional outcome after repeat surgery of tumor progression or treatment-associated changes in high-grade glioma: Clinical and radiological predictors

**DOI:** 10.1093/nop/npag002

**Published:** 2026-01-09

**Authors:** Anouk M de Jong, Maud E Kortman, Maarten W Kreek, Christina M Flies, Aline P Becker, Matthijs van der Meulen, Filip Y F L de Vos, Jan-Willem Dankbaar, Joost J C Verhoeff, Pierre A J T Robe, Tom J Snijders

**Affiliations:** Department of Neurology and Neurosurgery, UMC Utrecht Brain Center, University Medical Center Utrecht, Utrecht, The Netherlands; Department of Radiation Oncology, University Medical Center Utrecht, Utrecht, The Netherlands; Department of Neurology and Neurosurgery, UMC Utrecht Brain Center, University Medical Center Utrecht, Utrecht, The Netherlands; Department of Neurology and Neurosurgery, UMC Utrecht Brain Center, University Medical Center Utrecht, Utrecht, The Netherlands; Department of Neurology and Neurosurgery, UMC Utrecht Brain Center, University Medical Center Utrecht, Utrecht, The Netherlands; Department of Radiation Oncology, The Ohio State University College of Medicine, Columbus, Ohio, USA (A.P.B., P.A.J.T.R.); Department of Neurology, Leiden University Medical Center, Leiden, The Netherlands; Department of Medical Oncology, University Medical Center Utrecht, Utrecht, The Netherlands; Department of Radiology, University Medical Center Utrecht, Utrecht, The Netherlands; Department of Radiation Oncology, University Medical Center Utrecht, Utrecht, The Netherlands; Department of Radiation Oncology, Amsterdam University Medical Center, Amsterdam, The Netherlands; Department of Neurology and Neurosurgery, UMC Utrecht Brain Center, University Medical Center Utrecht, Utrecht, The Netherlands; Department of Radiation Oncology, The Ohio State University College of Medicine, Columbus, Ohio, USA (A.P.B., P.A.J.T.R.); Department of Neurology and Neurosurgery, UMC Utrecht Brain Center, University Medical Center Utrecht, Utrecht, The Netherlands

**Keywords:** functional outcome, high-grade glioma, pseudoprogression, radionecrosis, repeat surgery

## Abstract

**Background:**

Recurrence of high-grade glioma after primary treatment is inevitable. New contrast-enhancing lesions on follow-up MRI may indicate tumor progression, treatment-associated changes (TAC), or both. A repeated surgical procedure, either biopsy or resection, provides diagnostic clarity and therapeutic benefit, but carries risks. In the patient-specific decision to operate, current literature offers limited guidance on which patients may benefit. This study aimed to describe functional outcomes after repeat surgery of previously irradiated glioma, and to identify predictors of functional deterioration.

**Methods:**

Single-center retrospective cohort study. Adults with diffuse glioma who underwent first-line radiotherapy and subsequent repeat surgery (biopsy or resection) for suspected high-grade radiological progression were included. Lesions were histologically classified as progression, TAC, or both. The primary endpoint was functional outcome 30 days postoperatively, expressed as decline of Karnofsky Performance Status (KPS). Logistic regression identified predictors of KPS decline. Survival was assessed with Kaplan-Meier and Cox regression.

**Results:**

Of 166 patients, 37 (22.3%) experienced a KPS deterioration, 127 (76.5%) remained stable, and 2 (1.2%) improved. Rates of functional deterioration were similar after biopsies and resections. Preoperative steroid usage (odds ratio = 2.73, 95% confidence interval = 1.09-6.79, *P* = .031) was associated with KPS deterioration. Histological diagnosis was not associated with functional outcome (χ^2^, *P* = .361). Patients with deterioration had shorter median survival (4 vs 11 months, *P* < .001).

**Conclusions:**

In this cohort, 22.3% of patients experienced functional decline 30 days after a repeat surgery (biopsy or resection). Preoperative steroid usage predicted deterioration. These findings may provide clinical guidance in identifying which patients will benefit from repeat surgery.

Key Points

- 22.3% of high-grade glioma patients had a functional decline after repeat surgery
- Functional decline impacts survival and is associated with steroid use
- Risk of functional decline is not associated with histological outcome

Importance of the Study

A repeat surgery in high-grade glioma (HGG) patients with new contrast-enhancing lesions after radiotherapy is often considered to distinguish tumor progression from treatment-associated changes (TAC). Identifying which patients will benefit from the repeat surgery is challenging. Existing literature mainly focuses on surgical outcomes and offers limited information about functional outcomes or patient selection. Our study added to this literature by assessing functional outcomes and integrating these with detailed patient characteristics. This offers valuable guidance for clinicians when considering repeat surgery, either biopsy or resection, enabling better patient selection based on individual determinants. For example, patients receiving preoperative steroids should be counseled about the increased risk of functional deterioration. Patients with suspected TAC show similar functional outcomes to those with true tumor progression, indicating that histological diagnosis alone should not dictate surgical decisions. Importantly, functional decline after repeat surgery impacts subsequent adjuvant treatment and survival, highlighting the need to balance oncologic benefit while maintaining patient function.

High-grade gliomas are the most common primary malignant brain tumor in adults.^1^ These tumors have a variable, but generally poor prognosis and are often rapidly progressive.^2^ Despite extensive treatment, the median overall survival (OS) for patients with the most aggressive type of these tumors, glioblastoma (GBM, IDH-wildtype, WHO-grade 4 astrocytoma), is only 14 to 16 months.^3^ In contrast, survival rates for lower grade diffuse gliomas can extend to 10 years and longer.^4^ However, regardless of tumor grade, recurrence is currently inevitable, with approximately 90% of patients experiencing tumor regrowth within 2 years of the initial diagnosis.^5^^,^^6^

Primary treatment of a high-grade glioma usually consists of maximum safe resection, followed by radiotherapy with concomitant and/or adjuvant chemotherapy.^2^ After completing primary treatment, patients undergo routine follow-up MRIs. These scans can reveal new or increasing contrast-enhancing lesions, which may result from tumor progression, treatment-associated changes (TAC), or a combination of both.^7^

The most definitive method for distinguishing progression from TAC is a surgical intervention, with histopathological examination of the tissue, acquired from biopsy or resection, which provides the most certain diagnosis.^8^ Surgical resection is typically performed for presumed progressive disease, but in some cases, the lesion is later found to be a treatment-associated effect, such as radionecrosis or pseudoprogression.^9^ In these patients, surgery will not result in oncological (survival) benefit, but it may still reduce symptoms related to mass effect.^10^ Therefore, neurosurgical resection can have a therapeutic role: It has a cytoreductive effect in patients with either tumor progression or TAC, which may improve OS, by alleviating mass effect, which benefits all patients, including those with TAC.^11–13^ However, surgical intervention is invasive and the decision to perform biopsy or neurosurgical resection needs to be carefully weighed against the risks in a multidisciplinary meeting, particularly in this vulnerable patient population with limited life expectancy.^14^

Currently, identifying which patients with a possible recurrence will obtain net benefit from repeat surgery remains challenging. Identifying predictive factors for surgical benefit is crucial.^15^ One of the most predictive factors for benefit from a re-resection is the pretreatment Karnofsky Performance Status (KPS).^16^ KPS is a widely used scale to assess functional status in brain tumor patients and serves as a predictor of clinical outcomes, including survival.^17^ Other predictive factors of OS include the extent of disease, the histological grade (both primary tumor grade and the tumor grade at recurrence, including certain molecular findings such as MGMT methylation status), the relapse-free interval, and the recurrence pattern (local vs diffuse).^18–21^

Most existing literature regarding repeat surgeries focuses on surgical outcomes (such as extent of resection, mortality, and complications) rather than on functional outcomes (such as performance scales, changes in neurological status and quality of life).^22^ Furthermore, little is known whether functional outcome following repeat surgery is influenced by the underlying histological diagnosis: tumor progression or TAC.

In summary, the decision to perform a repeat surgery is patient-specific, and current literature offers limited guidance on which patients may benefit from the procedure.

This retrospective consecutive single-center cohort study aims to describe, in the domain of patients with a progressive radiological lesion in whom repeat surgery is considered, functional outcomes after such surgery (either biopsy or resection) and to identify potential determinants of functional deterioration. Specifically, the role of the histological diagnosis is investigated. We describe the impact of these outcomes on the oncological follow-up trajectory, including decisions regarding adjuvant treatment and subsequent survival outcomes.

## Methods

### Participants

All adult patients with a primary diffuse glioma (WHO grade 2-4), who underwent radiotherapy, developed a contrast-enhanced lesion on follow-up MRI at any timepoint postradiotherapy, and subsequently underwent a repeat surgery at the UMC Utrecht hospital between January 1, 2010, and May 31, 2019, were eligible for inclusion. The original histopathological diagnoses were updated according to the 2021 WHO classification by a medical researcher, a neuro-oncologist and a pathologist, based on molecular data available.

In patients who underwent more than 1 repeat surgery, we only analyzed the first repeat surgery.

### Data Collection

Clinical determinants were extracted from electronic patient records and were selected from known conventional presurgical risk factors^23^ and other previous literature.^24^^,^^25^ These included preoperative characteristics (such as age, medical history, family history, steroid usage and preoperative KPS [with cases where KPS <80 was classified as poor]), tumor characteristics (preoperative and postoperative histological diagnosis, including primary tumor grade, IDH-status, preoperative tumor size, mass effect, tumor location categorized by region/lobe), surgical characteristics (awake procedure, limitations during procedure and type of procedure/extent of resection), and postoperative characteristics (complications, KPS after 30 days and neurological symptoms after 30 days). KPS were copied from charts; if KPS was not given, we deduced the KPS from case notes, if sufficient information was available. No patients were excluded due to missing KPS. In addition, the indication for surgery was retrieved from medical records, as documented by the multidisciplinary tumor board. Reasons were categorized as diagnostic dilemma (TAC vs progression, low-grade vs high-grade), treatment of suspected progression, identification of new targets for adjuvant treatment, or mass reduction. Extent of resection was determined based on evaluation of postoperative MRI scans, and categorized according to the RANO-resect criteria.^12^ Systematic volumetric analysis was challenging due to heterogeneity in postoperative scan protocols, which often lacked 3D T1—post gadolinium. However, in patients with a small postoperative tumor remnant (for whom the RANO-resect category was not immediately clear), volumetric measurements were performed to allow accurate assignment of the RANO-resect category to all patients with available imaging.

Radiological determinants were selected based on literature^24^^,^^26^^,^^27^ and established in consultation with neurologists and neurosurgeons, drawing on their own experiences regarding which determinants influenced the neurological outcome. The determinants were assessed on the conventional follow-up MRI scan that prompted the decision to perform a repeat surgery. Conventional MRI included T1-weighted imaging (with and without gadolinium [gd] as contrast agent), T2-turbo spin echo, fluid-attenuated inversion recovery (FLAIR), and apparent diffusion coefficient (ADC) imaging. The following determinants were assessed: (1) the sum of products of diameters (SPD) of the gadolinium-enhanced tumor size according to RANO 2.0 criteria,^28^ (2) SPD of FLAIR/T2 enhancement, subdivided in (3) ependymal involvement on gadolinium-enhanced T1, (4) ependymal involvement on FLAIR/T2, (5) midline shift > 5 mm, (6) cyst involvement with a diameter >15 mm, (7) (left) thalamus involvement on T2-FLAIR, (8) hydrocephalus, compared with 2 previous scans and based on the radiologist’s report (9) diffusion restriction in the nonenhancing part of the tumor. Image assessment was performed by a medical student supervised by an experienced neuroradiologist. Supervision involved the first 10 scans were reviewed together, and 15 scans were also assessed by the neuroradiologist and used for interrater reliability. Difficult cases were discussed with the neuroradiologist. Interrater reliability was evaluated using Cohen κ and Pearson correlation coefficient (*r*). Images with examples of the assessment of the determinants can be found in the Supplementary Material (Figures S1 and S2).

### Outcomes

The primary outcome was the functional outcome following repeat surgery, assessed using the Karnofsky Performance Status(KPS), before repeat surgery and approximately 30 days after the surgery. Repeat surgery was categorized as (1) gross total resection, corresponding to RANO-resect category 2A or 2B, and (2) subtotal resection/debulking all corresponded to class 3A or 3B. Supramaximal resections were not performed in our center, as this is not a part of our institutional practice. Functional outcome assessments were conducted by either a neurosurgeon or a neuro-oncologist. A deterioration in functional outcome was defined as a decrease of 20 or more in the KPS score at ∼30 days, in line with recent literature on postoperative outcomes.^29^ KPS was also recorded at discharge, and to further understand our findings, we investigated the trajectory of KPS changes at both discharge and approximately 30 days after the resection. All patients, including those who underwent biopsy only, were included in all analyses. The functional outcome for patients who underwent a biopsy were additionally evaluated separately, given that no volume reduction was achieved, and compared with the patients who underwent a resection.

In addition to the previously mentioned main analysis, we performed a subgroup analysis, with patients with a preoperative KPS ≥ 70, as a score of 70 represents the threshold for independence in activities of daily living (ADL), whereas a lower score indicates the need for assistance. The primary survival outcome was OS, defined from the date of surgery to the date of death or last follow-up.

We assessed the tumor-directed treatments that patients received after the resection (adjuvant treatment), and we categorized these into radiotherapy, chemotherapy, and experimental treatment. The completion of these treatments was defined as follows: completion of radiotherapy was defined as receiving all planned radiation; completion of chemotherapy was defined as at least 6 cycles of temozolomide or at least 4 cycles of lomustine (with or without procarbazine and vincristine).

Histological outcomes included tumor progression and TAC. In this study, TAC were defined as posttherapy changes, including inflammatory infiltration, gliosis, hemorrhage, edema, radiation necrosis, or cellular senescence without evidence of mitotically active tumor. Tumor progression was defined as the presence of proliferating tumor (ie, conspicuous presence of mitotic activity with or without TAC).

### Statistical Analysis

The statistical analysis was performed with use of IBM SPSS Statistics, version 25. A *P* value of <.05 was considered statistically significant.

Descriptive statistics were used to summarize patient characteristics and key variables.

To identify possible preoperative clinical determinants of KPS deterioration, univariable logistic regression was executed. Univariable logistic analysis allowed for selection of variables to conduct multivariable logistic regression (*P* < .25). To understand the underlying mechanisms of KPS deterioration, a univariable logistic regression analysis was performed to correlate postoperative determinants (such as complications) to the outcome of KPS deterioration. Missing data were addressed with use of multiple imputation with the fully conditional specification method in SPSS, version 25. Ten imputed datasets were generated, and regression analyses were performed on each dataset with results pooled accordingly.

To evaluate the relationship between histological outcome and functional deterioration, χ^2^ tests were used.

Descriptive statistics were used to describe treatment completion patterns. Chi-square tests were used to assess associations between functional outcome and postresection treatment choices.

Survival analysis was conducted with the Kaplan-Meier method and log-rank test. In addition, Cox proportional hazards regression was performed to evaluate potential prognostic factors—including KPS deterioration, extent of resection, and primary tumor grade—for OS after surgery, and to investigate the independent effect of KPS deterioration. As there were no missing values in the included variables, complete case analysis was performed. Given the differences in biology and prognosis, we analyzed differences in patients with IDH-mutated tumors and patients with IDH-wildtype tumors.

No formal power calculation was performed, as this was an exploratory retrospective analysis with use of all available patients.

## Results

The electronic patient files of all consecutive patients (n = 190) with a diffuse glioma who received recurrence surgery at the University Medical Center in Utrecht between January 2010 and May 2019 were screened. Of these, 24 patients were excluded, because it was not their first repeat surgery.

### Baseline Characteristics

A total of 166 participants were included in the study, with 54 women (32.5%) and 112 men (67.5%), as shown in Table 1. The mean age of participants was 53.7 years. Regarding the diagnosis of the primary tumor, the most common types were glioblastoma (IDH-wildtype astrocytoma grade 4) (39.2%) and astrocytoma, WHO grade 4 (either IDH-mutant [6.0%] or not otherwise specified [19.3%]).

**Table 1.** Baseline characteristics of 166 patients with a high-grade glioma, who developed a contrast-enhancing lesion after radiotherapy.

Characteristics		Values
**Sex, n (%)**	Female	54 (32.5%)
	Male	112 (67.5%)
**Age, years**	Mean ± SD (min-max)	53.70 ± 12.19 (18-78)
**Diagnosis of WHO-classification primary tumor, n (%)**	Astrocytoma grade 2, NOS	8 (4.8%)
	Oligodendroglioma grade 2 and 3	19 (11.4%)
	Astrocytoma grade 2 and 3, IDH mutant	24 (14.5%)
	Astrocytoma grade 3, NOS	8 (4.8%)
	Astrocytoma grade 4, IDH mutant	10 (6.0%)
	Glioblastoma (IDH wild type astrocytoma grade 4)	65 (39.2%)
	Astrocytoma grade 4, NOS	32 (19.3%)
**Extent of resection, n (%)**	Supramaximal resection (RANO 1)	0
	Maximal CE resection (RANO 2)	40 (24.1%)
	Submaximal resection (RANO 3)	94 (56.6%)
	Biopsy (RANO 4)	23 (13.9%)
**Time since radiotherapy, months**	Median (Q1-Q3)	14 (7-32.5)
**Awake vs not-awake, n (%)**	Awake	48 (28.9%)
	Not awake	118 (71.1%)
**Preoperative steroid use, n (%)**	Did not use steroids preoperatively	91 (54.8%)
	Low dose steroids (≤4 mg/d)	53 (32.0%)
	High dose steroids (>4 mg/d)	22 (13.0%)

Abbreviations: CE, contrast-enhanced; IDH, isocitrate dehydrogenase; NOS, not otherwise specified; 1p19q, chromosomal codeletion of 1p and 19q; SD, standard deviation; WHO, World Health Organization.

Regarding the extent of the resection, 23 participants (13.9%) underwent biopsy, 94 participants (56.6%) had a submaximal resection, and 40 participants (24.1%) underwent maximal CE resection. There were no supramaximal resections, as this is not part of our clinical practice. For 48 patients (28.9%), the surgery was executed in an awake-setting, while for 118 patients (71.1%), it was a nonawake procedure.

As for the indications for re-resection, 71 patients (42.8%) underwent surgery due to a diagnostic dilemma of treatment effect versus progression, and 7 patients (4.2%) because of uncertainty between low-grade and high-grade disease. Surgery was performed to treat suspected progression in 90 patients (54.2%), to identify new surgical targets in 17 patients (10.2%), and to achieve mass reduction due to symptoms in 31 patients (18.7%). As multiple indications could apply to a single patient, frequencies exceed 100%.

### Functional Outcome

Of the 166 eligible patients, both a preoperative and postoperative KPS were available. Thirty-seven patients (22.3%) showed a KPS deterioration of 20 or more. In 127 patients (76.5%), KPS remained stable. Two patients (1.2%) showed a KPS improvement of 20 or more.

### ADL Independence

Among the 152 patients with a preoperative KPS of 70 or higher (indicating ADL independence), 33 (21.7%) deteriorated to a KPS lower than 70, corresponding to loss of ADL independence.

### Trajectory of KPS Changes

**Figure 1.**
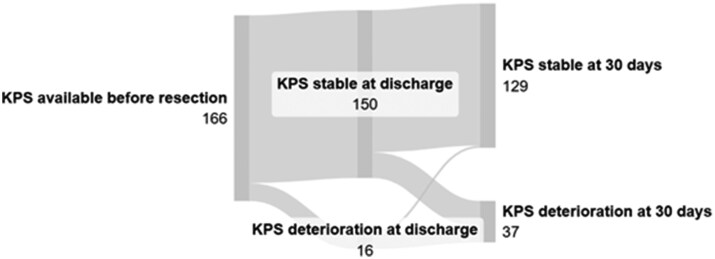
Sankey diagram depicting the trajectory of KPS in patients with high-grade glioma, who developed a contrast-enhancing lesion after radiotherapy and subsequently underwent repeat surgery. The diagram illustrates changes in KPS from baseline (before surgery) to discharge (stable/deterioration) and 30 days after surgery (stable/deterioration). Abbreviation: KPS, Karnofsky performance status.

To further understand the timing of functional deterioration, the trajectory of KPS changes was explored at 2 timepoints: at discharge and 30 days postresection. The trajectory of these changes is depicted in Figure 1. Of the 37 patients with deterioration at 30 days, the majority (62.2%) was still stable at discharge, suggesting deterioration at a later stage, possibly due to tumor progression.

### Preoperative Determinants

Univariable logistic regression of preoperative clinical determinants showed that preoperative steroid usage was significantly associated with KPS deterioration (odds ratio [OR] = 3.29, 95% confidence interval [CI] = 1.52-7.14, *P* = .003), as shown in Table 2. In addition, KPS deterioration occurred more frequently in patients whose primary tumor was IDH wild type (20 of 66, 30.3%) compared with those whose primary tumor was IDH-mutated (7 of 53, 13.2%), OR = 2.86, 95% CI = 1.10-7.41, *P* = .031). Other factors, such as diabetes, hypertension, left ventricular hypertrophy, smoking status, preoperative KPS and BMI, were not associated with KPS deterioration.

**Table 2.** Univariable and multivariable analysis of preoperative clinical and radiological determinants associated with functional outcome following repeat surgery.

KPS difference	KPS stable/improvement (n = 129)^a^	KPS deteriorated (n = 37)^a^	Univariable OR (95% CI)^b^	*P* value	Multivariable OR (95% CI)^b^	*P* value
***Preoperative clinical determinants***						
**Diabetes: Y, n (%)**	4 (3.1%)	4 (10.8%)	3.79 (0.90-15.96)	.069	2.41 (0.51-11.28)	.266
**Hypertension: Y, n (%)**	23 (17.8%)	9 (24.3%)	1.48 (0.62-3.56)	.379		
**Left ventricular hypertrophy: Y, n (%)**	11 (8.9%)	4 (11.4%)	1.31 (0.37-4.61)	.671		
**Smoking:**						
** Never smoked, n (%), ref**	71 (55.0%)	23 (63.9%)				
** Former smoker, n (%)**	35 (27.1%)	12 (33.3%)	1.07 (0.48-2.40)	.869	1.17 (0.48-2.88)	.735
** Active smoker, n (%)**	23 (17.8%)	1 (2.8%)	0.18 (0.02-1.31)	.090	0.16 (0.2-1.27)	.082
**BMI, mean ± SD**	25.62 ± 4.33	25.47 ± 3.92	0.99 (0.91-1.08)	.848		
**OSAS: Y, n (%)**	1 (0.8%)	0	n.a.	n.a.		
**Atrial fibrillation: Y, n (%)**	5 (4.0%)	3 (8.1%)	1.76 (0.41-7.48)	.446		
**Preop steroid usage: Y, n (%)**	50 (38.8%)	25 (67.6%)	3.29 (1.52-7.14)	**.003**	2.73 (1.09-6.79)	.031
**Preop bevacizumab usage: Y**	0	1 (2.8%)	n.a.	n.a.		
**Preop KPS < 80: Y, n (%)**	31 (24.0%)	13 (35.1%)	1.71(0.78-3.76)	.180	1.21 (0.48-3.06)	.691
**IDH status primary tumor**						
** IDH-mutation, n (%), ref**	46 (35.7%)	7 (18.9%)				
** IDH wild type, n (%)**	46 (35.7%)	20 (54.1%)	2.86 (1.10-7.41)	**.031**	2.00 (0.70-5.73)	.199
** NOS, n (%)**	37 (28.7%)	10 (27.0%)	1.78 (0.62-5.12)	.287	1.12 (0.34-3.66)	.857
***Preoperative radiological determinants***						
**SPD tumor size (mm^2^): mean ± SD**	901.59 ± 820.03	1226.23 ± 1086.77	1.000307 (0.999915-1.000699)	0.124	1.000059 (0.999541-1.000576)	.823
**SPD FLAIR enhancement (mm^2^): mean ± SD**	3479.20 ± 2004.05	3603.71 ± 2508.56	1.000043 (0.999871-1.000215)	0.622		
**Ependymal involvement Gd enhancement: Y, n (%)**	64 (54.2%)	25(67.6%)	1.70 (0.76-3.81)	0.201	1.46 (0.58-3.67)	.421
**Ependymal involvement FLAIR enhancement: Y, n (%)**	111 (94.1%)	36 (97.3%)	3.92 (0.47-32.88)	0.207	1.95 (0.18-21.17)	.583
**Midline shift: Y, n (%)**	18 (15.1%)	7 (18.9%)	1.09 (0.41-2.92)	0.867		
**Cyst involvement: Y, n (%)**	32 (26.9%)	10 (27.0%)	1.03 (0.44-2.39)	0.949		
**Thalamus involvement: Y, n (%)**	34 (29.1%)	17 (45.9%)	1.94 (0.91-4.16)	0.088	1.63 (0.64-4.17)	.309
**Hydrocephalus: Y, n (%)**	2 (1.7%)	1(2.8%)	0.97 (0.08-11.22)	0.982		
**Diffusion restriction in non-enhancing tumor: Y, n (%)**	12 (11.0%)	7(20.0%)	1.28 (0.48-3.41)	0.623		

Abbreviations: CI, confidence interval; FLAIR, fluid-attenuated inversion recovery; Gd, gadolinium; KPS, Karnofsky Performance Status; n, number; OR, odds ratio; OSAS, obstructive sleep apnea syndrome; ref, reference category; SD, standard deviation; SPD, sum of the products of perpendicular diameters; Y, yes.

Bold values indicate statistically significant associations.

a Absolute frequencies were based on original (non-imputed) data.

b OR, 95% CI, and *P* values are based on univariable logistic regression after multiple imputation (10 datasets), pooled using Rubin rules.

Univariable logistic regression of preoperative radiological determinants, and by brain region/location, showed no significant association with KPS deterioration.

Univariable logistic analysis allowed for selection of variables to conduct multivariable logistic regression (*P* < .25). Variables on brain regions/lobes were not included in multivariable analysis (also those with *P* of .06-.25) because of their collinearity. The following variables were included: preoperative steroid usage, preoperative KPS < 80, diabetes, smoking status, IDH-mutation status of the primary tumor, SPD tumor size, ependymal involvement Gd enhancement, ependymal FLAIR enhancement, and thalamus involvement. The multivariable logistic regression results indicated that preoperative steroid usage (OR = 2.73, 95% CI = 1.09-6.79, *P* = .031) was independently associated with KPS deterioration, as shown in Table 2.

### Postoperative Determinants

To understand the nature of KPS deterioration, a univariable logistic regression analysis was performed on postoperative determinants, as shown in Table 3. Altered consciousness (defined as a Glasgow Coma Scale [GCS] < 15) was related to KPS deterioration (OR = 16.84, 95% CI = 4.31-65.75, *P* < .001), as well as new motor deficit (OR = 5.31, 95% CI = 2.35-12.00, *P* < .001), new cranial nerve deficit (OR = 3.75, 95% CI = 1.60-8.80, *P* = .002), and new language disorder (OR = 3.28, 95% CI = 1.41-7.66, *P* = .006). A complete discontinuation of postoperative steroid usage was associated with a reduced risk of KPS deterioration (OR = 0.39, 95% CI = 0.16-0.93, *P* = .033). Other variables, including cognitive changes, did not show a significant association with KPS deterioration.

**Table 3.** Univariable logistic regression of postoperative determinants associated with functional outcome following repeat surgery.

KPS difference	KPS stable/improvement (n = 129)^a^	KPS deteriorated (n = 37)^a^	OR (95%CI)^b^	*P* value
**Postop steroid usage: Y, n (%)**	109 (85.2%)	35 (94.6%)	3.08 (0.68-13.88)	.143
**Postop steroid usage completely discontin ued: Y, n (%)**	64 (67.4%)	12 (44.4%)	0.39 (0.16 - 0.93)	**.033**
**Altered consciousness (GCS < 15): Y, n (%)**	3 (2.3%)	10 (28.6%)	16.84 (4.31-65.75)	**<.001**
**New sensibility loss: Y, n (%)**	14 (10.9%)	5 (14.3%)	1.49 (0.50-4.44)	.477
**New cranial nerve deficit: Y, n (%)**	18 (14.0%)	13 (37.1%)	3.75 (1.60-8.80)	.002
**New motor deficit: Y, n (%)**	19 (14.7%)	16 (45.7%)	5.31 (2.35-12.00)	**<.001**
**New language disorder: Y, n (%)**	19 (14.7%)	12 (34.3%)	3.28 (1.41-7.66)	**.006**
**New symptomatic seizure (after 7 days, within 30 days): Y, n (%)**	13 (10.1%)	4 (11.4%)	1.34 (0.39-4.55)	.641
**Cognitive change: Y, n (%)**	24 (18.6%)	9 (25.7%)	1.62 (0.68-3.87)	.280

Abbreviations: CI, confidence interval; GCS, Glasgow Coma Scale; KPS, Karnofsky Performance Status; n, number; OR, odds ratio; Postop, postoperatively; Y, yes.

Bold values indicate statistically significant associations.

a Absolute frequencies were based on original (non-imputed) data.

b OR, 95% CI, and *P* values are based on univariable logistic regression after multiple imputation (10 datasets), pooled using Rubin rules.

### KPS and Histological Outcome

A Pearson χ^2^ test (χ^2^ = 0.835, *P* = .361) revealed no significant association between histological outcome and KPS. Among patients with TAC, 26 (83.9%) had a stable or improved KPS, while 5 (16.1%) showed deterioration. In patients with progressive disease, 103 (76.3%) had stable or improved KPS, and 32 (23.7%) deteriorated.

Stratification by IDH status revealed that, among patients with IDH-mutant tumors, histological outcome was not significantly associated with KPS change. In contrast, in patients with IDH-wildtype tumors, KPS deterioration was significantly more frequent in those with progressive disease (19 of 52, 36.5%) than in those with TAC (1 of 14, 7.1%; Fisher exact *P* = .048).

### Biopsy Versus Resection

Among the 23 patients who received a biopsy, 18 (78.3%) remained stable or improved in KPS at 30 days. Five patients (21.7%) experienced deterioration: 1 patient showed an immediate postoperative decline, due to initiation of palliative sedation following the pathology result, and the other 4 patients showed delayed deterioration. These patients all had an MRI showing increasing edema or other signs of tumor progression. Multivariable logistic regression analysis, excluding the biopsy patients, yielded the same results, with only steroid usage (OR = 3.43, 95% CI = 1.20-9.81, *P* = .022) as a significant predictor of functional outcome, as shown in Table S4.

For the biopsy patients, among patients with TAC, 11 (100%) had a stable or improved KPS, while none showed deterioration. In contrast, among patients with progressive disease, 7 (58.3%) had stable or improved KPS, and 5 (41.7%) deteriorated (Fisher Exact *P* = .037).

For the patients who underwent a resection, no significant association between functional and histological outcome was observed. Among patients with TAC, 15 (75.0%) had a stable or improved KPS, while 5 (25.0%) showed deterioration. In patients with progressive disease, 92 (80.7%) had a stable or improved KPS, while 22 (19.3%) showed deterioration (Fisher Exact *P* = .553).

### Retreatment After Re-Resection

Of 135 patients who had histopathological confirmation of true progression, 103 patients (76.3%) received antineoplastic treatment after the re-resection. Twelve patients (11.7%) received re-irradiation, 72 (69.9%) chemotherapy, and 19 (18.4%) experimental treatment. In 2 patients, data on further treatment were missing.

Of the 103 patients that received treatment, only 44 completed treatment (42.7%) Fifty patients, all treated with chemotherapy or experimental treatment, did not complete the second treatment due to progression (41, 82.0%), toxicity (5, 10.0%), other disease (1, 2.0%), or due to unknown reasons (3, 6.0%).

**Figure 2.**
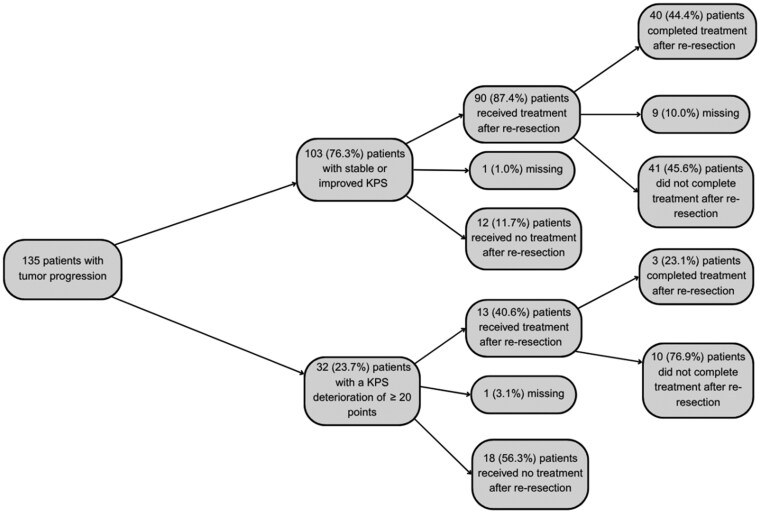
Flowchart of functional outcomes, treatment patterns, and completion rates following repeat surgery in patients with high-grade glioma and a contrast-enhancing lesion after radiotherapy, with histopathology confirming true tumor progression or a mixed lesion (TAC excluded). A deterioration in functional outcome was defined as a decrease of ≥20 points in Karnofsky Performance Status (KPS), measured before resection and approximately 30 days after resection. Abbreviation: TAC, treatment-associated changes.

Treatment patterns after re-resection are given in Figure 2. Within the patients with progression, those with KPS deterioration received treatment less often than those with stable or improved KPS (41.9% vs 88.2%, χ^2^ = 29.175, *P* < .001). A Pearson χ^2^ test (χ^2^ = 3.413, *P* = .065) did not show a significant difference between the 2 groups in terms of treatment completion. Twelve patients with a stable or improved KPS (11.8%) did not receive treatment after re-resection. Reasons for this include secondary deterioration after 30 days, patient wishes, or lack of indication for additional treatment (particularly in cases where the resection specimen showed only a minimal component of high-grade progression).

### Survival Analysis

Overall survival of the total cohort was assessed using Kaplan-Meier analysis. The median OS was 10 months (95% CI = 7.81-12.19). When stratified by IDH-mutation status, median OS was 21.0 (95% CI = 9.79-32.21), and 8.0 months (95% CI = 6.67-9.33) for IDH-wildtype patients (log-rank *P* < .001).

A Kaplan-Meier survival analysis was performed to assess OS, stratified by KPS deterioration versus KPS stable/improvement. Median survival in the KPS deterioration group was 4 months (95% CI = 2.21-5.79), whereas median survival for the KPS stable/improvement group was 11 months (95% CI = 8.58-13.42) (log-rank test 29.558, *P* < .001).

**Figure 3.**
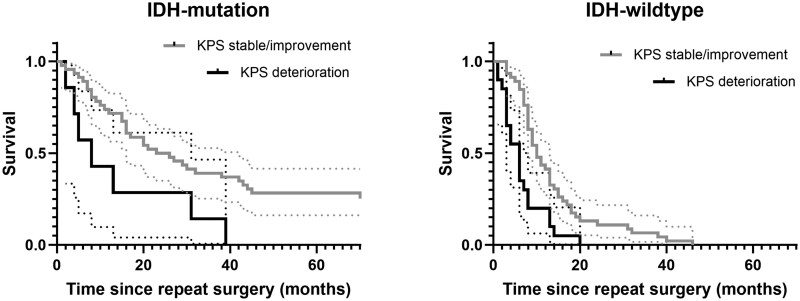
Kaplan-Meier curves for overall survival of patients with high-grade glioma who developed a contrast-enhancing lesion after radiotherapy and subsequently underwent repeat surgery, stratified by functional outcome based on the Karnofsky Performance Status (KPS) before and ∼30 days after re-resection, shown separately for IDH-mutant tumors (left) and IDH-wildtype tumors (right).

Median survival was shorter in the KPS deterioration group than for the KPS stable/improvement group, both for IDH mutant (8 months [95% CI = 0.3-15.7] vs 23 months [95% CI = 11.6-34.4], log-rank *P* = .016) and for IDH-wildtype tumors (6 months [95% CI = 3.9-8.1] vs 10 months [95% CI = 7.8-12.2], log-rank *P* < .001), see Figure 3.

A Kaplan-Meier analysis to assess OS in relation to postoperative KPS (30 days after re-resection) showed a significant association between KPS and survival. The median survival for patients with a postoperative KPS of 70 or lower was 6 (95% CI = 4.30-7.70) and 12 months for patients with a postoperative KPS of 80 or higher (95% CI = 9.46-14.54) (log-rank test 7.921, *P* = .005).

A Cox regression analysis showed a significant effect from KPS deterioration on survival, where patients with a KPS deterioration had a higher risk of dying than patients with a stable/improved KPS (hazard ratio [HR] = 2.66, *P* < .001). Other included covariates (extent of resection, histological outcome, and primary tumor grade) also showed significant effects, of which the directions are consistent with prior literature. The results of the Cox regression analysis can be seen in Table S1.

## Discussion

This retrospective consecutive single-center cohort study aimed to describe functional outcomes after re-resection, based on Karnofsky Performance Status (KPS), and to identify potential determinants of functional deterioration. In this cohort, most patients remained stable (76.5%), and a substantial minority of patients either deteriorated (KPS decrease 20 or more, in 22.3%) or improved (KPS increase of 20 or more, in 1.2%).

Our additional analysis of the timing of the functional deterioration revealed that over half of the patients with deterioration (62.2%) showed delayed deterioration after hospital discharge, while only 9.6% of the total cohort deteriorated directly postoperatively. This implies that a large portion of postoperative decline may not be attributable to immediate surgical factors, but could be driven by progressive disease or other delayed mechanisms. This notion is further supported by the observation that 22% of patients who underwent biopsy only still experienced functional deterioration, probably associated with tumor progression. However, given the retrospective nature of this study and absence of systematic postoperative imaging at 30 days, we cannot definitively distinguish between these potential causes.

Our results show that patients with IDH-wildtype tumors had a higher rate of KPS deterioration in univariable analysis, compared with patients with IDH-mutated tumors. However, when adjusting for preoperative clinical and tumor-related factors, this association was no longer statistically significant, suggesting that the effect of IDH-status on functional outcome is partly mediated by these other factors, such as preoperative steroid usage.

Preoperative steroid usage (odds ratio [OR] = 2.73, *P* = .031) was a significant predictor of KPS deterioration in univariable and multivariable analysis. This suggests that this preoperative factor significantly contributes to the risk of postoperative functional deterioration. Further explanatory analysis revealed that the deterioration of KPS in this cohort was primarily associated with altered consciousness, new motor deficit, new cranial nerve deficit, and language disorders, while complete discontinuation of postoperative steroid usage emerged as a protective factor.

In patients with glioma, corticosteroids are used to manage neurological symptoms caused by vasogenic peritumoral edema.^30^^,^^31^ However, despite their benefits in their symptomatic treatment, steroids are associated with numerous side effects. These side effects include hyperglycemia, insulin resistance, immunosuppression, delayed wound healing, behavioral changes, and venous thromboembolism.^32^ Previous studies showed a negative impact of preoperative steroid usage on survival^33^^,^^34^ but did not investigate the association of steroid usage with postoperative functional outcome. However, Link et al.found that perioperative hyperglycemia is associated with postoperative neurological deterioration in patients with glioblastoma.^35^ This suggests that hyperglycemia caused by preoperative steroid usage might be explanatory for our found risk of KPS deterioration. Also steroid usage might inhibit the antitumor immune response in glioma.^36^ During a repeat surgery of tumor progression, this might lead to a poorer prognosis and explain a worse functional outcome.

The median OS in our cohort was 8 months for patients with IDH-wildtype tumors. This is comparable with existing literature, with ranges of OS after reoperation from 4.6 to 9 months,^37–46^ reviewed by Hervey-Jumper et al.^47^ The median OS was 21.0 months for patients with IDH-mutant tumors, placing our survival data at the favorable end of the known spectrum for recurrent IDH-mutant tumors.^48^ Our findings indicate that KPS deterioration is associated with significantly poorer survival outcomes both for IDH-mutant and IDH-wildtype tumors. The median survival for the IDH-mutant group was 8 months in the KPS deterioration group and 23 months for those with stable or improved KPS (*P* = .016). For IDH-wildtype tumors, the median survival was, respectively, 6 and 10 months (*P* < .001). The association of KPS deterioration and poor survival remains significant in a multivariable Cox regression analysis adjusting for type of surgical intervention and tumor grade, consistent with published favorable prognostic effect of resection (vs biopsy) and IDH-mutations.^18^^,^^19^^,^^21^ These results emphasize the importance of KPS as a key prognostic factor. Notably, patients experiencing KPS deterioration were less likely to receive subsequent antineoplastic treatment after the re-resection, which may partly contribute to their shorter observed survival, and as such, forms a source of potential bias.

In our study, no overall significant association between histological outcome and KPS was found (except for those who underwent a biopsy). Approximately 20% of our patients had TAC rather than tumor progression, a percentage that may be higher than typically anticipated. There is a theoretical concern that TAC, such as radiation necrosis, may be more diffusely distributed and interspersed with functional brain tissue compared with vital tumor, potentially increasing the risk of perioperative morbidity. However, we observed that patients with TAC experienced functional outcomes, comparable with those with histologically confirmed tumor progression. Stratified analysis by IDH status showed that, for patients with IDH-mutant tumors, the histological result from the repeat surgery was not significantly associated with functional outcome, whereas for patients with IDH-wildtype tumors, KPS deterioration was more frequent in those with progressive disease than in those with TAC. These findings suggest that although TAC are not uncommon and should be considered in the differential diagnosis when resecting contrast-enhancing lesions, their presence does not appear to negatively influence postoperative functional status. Therefore, when counseling patients regarding a re-resection, the possibility of TAC should be discussed, but it may not necessitate a different approach regarding expected functional outcomes.

We included both patients with a biopsy and a re-resection, to reflect the full range of patients undergoing a surgical procedure for a progressive glial lesion. Although the subpopulations of biopsy patients and re-resection patients showed some notable differences, they were similar regarding the rate and determinants of functional deterioration.

This study has notable strengths. The large, consecutive patient cohort of 166 patients enhances the reliability of the findings. Moreover, the integration of functional outcome data with detailed patient characteristics and histopathological data—including the classification into TAC and progressive tumor—provides valuable insights to existing literature.

This study has several limitations, inherent to its retrospective design. Potential biases such as residual confounding cannot be fully excluded. The absence of an independent validation cohort may limit the generalizability of these findings. These factors should be considered when interpreting the results. Of note, the study sample forms a selection based on the indication for re-resection. This indication is made based on the local hospital’s standards, which may limit generalizability. However, within this selected group, all consecutive patients that met the inclusion criteria were included, so no additional selection bias seems to have occurred. The relation between steroid usage and KPS deterioration could be confounded by several factors. Steroid usage could be a marker of infiltrative and/or more aggressive tumor tissue (indeed, preoperative steroid use is more common in IDHwt tumors than IDHmut) or general medical condition. This complicates the interpretation of the effect of steroid usage on KPS deterioration. Furthermore, the KPS is an instrumental variable, with its own limitations, and it was only assessed at 30 days, which may not fully capture any long-term functional outcomes. Specifically, change in KPS may miss more subtle changes in functioning and is limited by ceiling effects in patients with high KPS at baseline. The quality of available imaging of this heterogeneous population made complete volumetric analysis challenging. Despite this limitation, it was possible to provide extent-of-resection data according to RANO-resect for all patients. Finally, there are no data on quality of life or cognition.

Limited literature is available, describing patient characteristics of patients with an irradiated diffuse glioma, undergoing a repeat surgery. The current cohort confirms the variability in functional outcome. Despite our center being a large academic neuro-oncology center, as a mono-center study, the results are a reflection of local practice. Our findings can still offer insights into what may determine a patient’s outcome and can also offer valuable guidance for clinicians when they consider repeat surgery, based on specific patient characteristics. Patients receiving preoperative steroids should be counseled on the potential risk of deterioration of preexisting symptoms.

However, further evidence from multicenter cohort studies or a randomized controlled trial is required to conclusively confirm our findings and enhance their generalizability. One relevant aspect could be the role of tumor location. In our current analysis, we did not find an association between involvement of certain (lobe-based) brain regions and risk of functional deterioration; investigating the role of tumor location more thoroughly could provide insights into region-specific vulnerabilities and their impact on postoperative recovery. In addition, a more detailed analysis of radiological characteristics may help distinguish true tumor progression from TAC, improving patient selection for surgery and avoiding surgery in cases with TAC without significant mass effect. Both advanced MRI and nuclear techniques can be helpful.^49^ The influence of tumor molecular profiles on functional outcomes and response to treatment also warrants further exploration. Finally, comparing outcomes between awake and nonawake surgery could help determine the optimal surgical approach for maximizing patient safety and preserving neurological function.

## Conclusions

In patients with a previously irradiated diffuse glioma undergoing a first repeat surgery (either biopsy or resection), a clinically relevant deterioration after re-resection occurred in 22.3% of all patients. A possible determinant of this deterioration is preoperative steroid usage. The KPS deterioration is associated with poorer survival. In cases with a histological diagnosis of TAC such as radionecrosis, functional outcome was comparable with outcome in patients with progression, suggesting that the surgical procedure benefits patient survival in both histological subgroups by reducing local and/or global mass effect. These data may offer guidance to patients, their proxies, and neurosurgical teams in perioperative decision-making.

## Supplementary Material

npag002_Supplementary_Data

## Data Availability

All data in this manuscript will be made available upon reasonable request.
