# Supplementary material for: Functional outcome after repeat surgery of tumor progression or treatment-associated changes in high-grade glioma: Clinical and radiological predictors
Source: Neurooncol Pract. 2026 Jan 9;13(4):673–84. doi: 10.1093/nop/npag002 (PMC13365144; doi:10.1093/nop/npag002)
Supplement: npag002_Supplementary_Data [file npag002_supplementary_data.docx]

# Supplementary material

*Supplementary Table 1. Cox regression analysis of functional outcome following repeat surgery*

| **Covariate** | **HR (95% CI)** | **P-value** |
| --- | --- | --- |
| **KPS deterioration vs KPS stable/improvement** | 2.66 (1.71 – 4.13) | <0.001 |
| **Extent of resection** |  |  |
| **Maximal resection vs biopsy** | 0.27 (0.13 – 0.53) | <0.001 |
| **Submaximal resection vs biopsy** | 0.40 (0.21 – 0.77) | 0.006 |
| **Biopsy, ref** |  |  |
| **Primary tumor grade** |  |  |
| **IDH mutation (ref)** |  |  |
| **IDH wild-type vs IDH mutation** | 2.64 (1.66 – 4.20) | <0.001 |
| **Not otherwise specified vs IDH mutation** | 2.24 (1.39 – 3.61) | <0.001 |

*Abbreviations: KPS = Karnofsky Performance Score, HR = Hazard Ratio, CI = Confidence Interval*

*Supplementary Table 2. Univariable logistic regression of post-operative complications associated with functional outcome following repeat surgery.*

| **KPS Difference** | **KPS Stable/Improvement**  **(n = 129)*^a^*** | **KPS Deteriorated**  **(n=37) *^a^*** | **OR (95% CI) *^b^*** | **P-value** |
| --- | --- | --- | --- | --- |
| **Wound infection: Y n (%)** | 10 (7.8%) | 9 (25.0%) | 3.99 (1.48 – 10.79) | **0.006** |
| **Wound leakage: Y n(%)** | 32 (24.8%) | 12 (32.4%) | 1.46 (0.66 – 3.23) | 0.356 |
| **Intracranial hemorrhage: Y n(%)** | 11(8.6%) | 6 (16.2%) | 2.08 (0.71 – 6.06) | 0.181 |
| **Ischemia: Y n(%)** | 32 (25.0%) | 10 (27.0%) | 1.16 (0.51 – 2.67) | 0.725 |
| **Edema: Y n(%)** | 10 (7.8%) | 9 (24.3%) | 3.71 (1.38– 9.98) | **0.009** |
| **Increased intracranial pressure: Y n(%)** | 7 (5.5%) | 8 (21.6%) | 4.42 (1.49-13.05) | **0.007** |
| **Acute symptomatic seizure within 7 days: Y n(%)** | 4 (3.1%) | 3 (8.1%) | 2.76 (0.59 – 12.92) | 0.198 |

1. *Absolute frequencies were based on original (non-imputed) data.*
2. *OR, 95% CI and p-values are based on univariable logistic regression after multiple imputation (10 datasets), pooled using Rubin’s rules.*

*Abbreviations: KPS = Karnofsky Performance Score, n = number, OR = Odds Ratio, CI = Confidence Interval, Y = Yes*

*Supplementary Table 3. Univariable logistic regression of operative variables associated with functional outcome following repeat surgery*

| **KPS Difference** | **KPS Stable/Improvement (n = 129) *^a^*** | | **KPS Deteriorated (n=37) *^a^*** | | **OR (95%CI) *^b^*** | | **P-value** | |
| --- | --- | --- | --- | --- | --- | --- | --- | --- |
| **Awake surgery: Y n(%)** | 38 (29.5%) | | 10 (27.0%) | | 0.86 (0.38 – 1.94) | | 0.706 | |
| **Surgery limited by function: Y n(%)** | 35 (92.1%) | | 9 (90.0%) | | 0.75 (0.07 – 8.09) | | 0.813 | |
| **Extent of resection, n (%)** | |  | |  | |  | |  |
| **Maximal resection** | | 32 (25.6%) | | 8 (25.0%) | | 0.94 (0.27 – 3.30) | | 0.919 |
| **Submaximal resection** | | 75 (60.0%) | | 19 (59.4%) | | 1.08 (0.36 – 3.24) | | 0.892 |
| **Biopsy, ref** | | 18 (14.4%) | | 5 (15.6%) | |  | |  |

1. *Absolute frequencies were based on original (non-imputed) data.*
2. *OR, 95% CI and p-values are based on univariable logistic regression after multiple imputation (10 datasets), pooled using Rubin’s rules.*

*Abbreviations: KPS = Karnofsky Performance Score, n = number, OR = Odds Ratio, CI = Confidence Interval, Y = Yes*

*Supplementary Table 4. Multivariable logistic regression of of pre-operative clinical and radiological determinants associated with functional outcome following repeat surgery (resection only, biopsy patients excluded)*

| **KPS Difference** | **KPS Stable/Improvement (n = 103) *^a^*** | | **KPS Deteriorated (n=27) *^a^*** | | **Multivariable OR (95%CI) *^b^*** | | **P-value** | |
| --- | --- | --- | --- | --- | --- | --- | --- | --- |
| **Diabetes: Y n (%)** | 4 (3.7%) | | 3 (11.1%) | | 2.11 (0.44 – 10.20) | | 0.355 | |
| **Smoking:** | |  | |  | |  | |  |
| **Never smoked n (%), ref** | | 62 (57.9% | | 19 (70.4%) | |  | |  |
| **Former smoker n (%)** | | 24 (22.4%) | | 7 (25.9%) | | 1.78 (0.65 – 4.89) | | 0.266 |
| **Active smoker n (%)** | | 21 (19.6%) | | 1 (3.7%) | | 0.14 (0.02 – 1.23) | | 0.077 |
| **Pre-op steroid usage n (%)** | | 42 (39.3%) | | 19 (70.4%) | | 3.43 (1.20 – 9.81) | | **0.022** |
| **Pre-op KPS < 80: Y n (%)** | | 28 (26.2%) | | 8 (29.6%) | | 1.09 (0.38 – 3.15) | | 0.875 |
| **IDH status primary tumor** | |  | |  | |  | |  |
| **IDH-mutation, n (%), ref** | | 38 (35.5%) | | 5 (18.5%) | |  | |  |
| **IDH wild type, n (%)** | | 40 (37.4%) | | 16 (59.3%) | | 2.14 (0.64 – 7.14) | | 0.216 |
| **NOS, n (%)** | | 29 (27.1%) | | 6 (22.2%) | | 1.31 (0.35 – 4.91) | | 0.692 |
| **SPD tumor size (mm^2^): Mean ± SD** | | 923.59 ± 830.85 | | 1212.38 ± 1001.26 | | 0.999960 (0.999289 – 1.000631) | | 0.906 |
| **Ependymal involvement Gd enhancement: Y n(%)** | | 51 (52.0%) | | 18 (66.7%) | | 1.65 (0.62 – 4.36) | | 0.315 |
| **Thalamus involvement: Y n(%)** | | 28 (28.9%) | | 11 (40.7%) | | 1.42 (0.48 – 4.16) | | 0.523 |
| **Ependymal FLAIR** | |  | |  | | n.a. | | n.a. |

1. *Absolute frequencies were based on original (non-imputed) data.*
2. *OR, 95% CI and p-values are based on univariable logistic regression after multiple imputation (10 datasets), pooled using Rubin’s rules.*

*Abbreviations: KPS = Karnofsky Performance Score, OR = Odds Ratio, CI = Confidence interval, SD = Standard Deviation, n = number, Y = Yes, ref = reference category, SPD = Sum of the Products of perpendicular Diameters, FLAIR = Fluor-Attenuated Inversion Recovery, Gd = Gadolinium*

*Supplementary Figure 1. Radiological determinants used in univariable analysis (1/2)
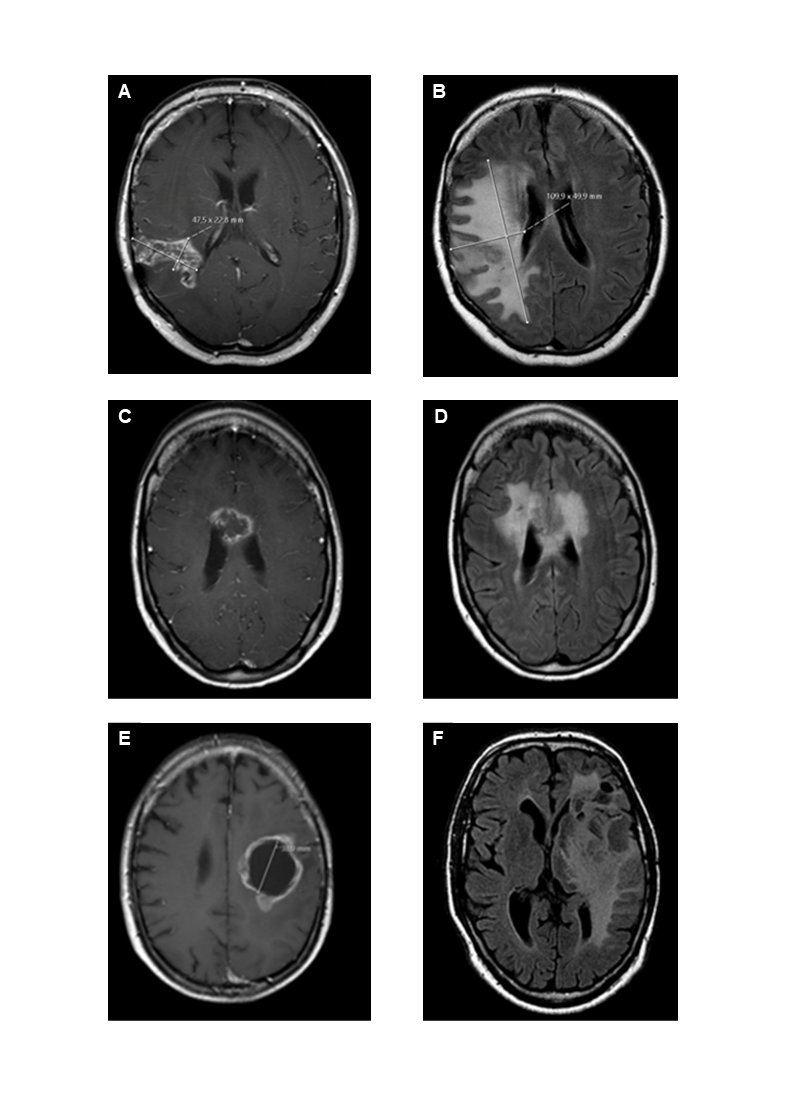
*

***The following examples of radiological determinants are shown****: SPD gadolinium (A), SPD FLAIR/T2 (B), ependymal involvement gadolinium (C), ependymal involvement FLAIR/T2 (D), cyst with diameter > 15 mm (E), and (left) thalamus involvement (T2/FLAIR) (F).*

*Supplementary Figure 2. Radiological determinants used in univariable analysis (2/2)*

*
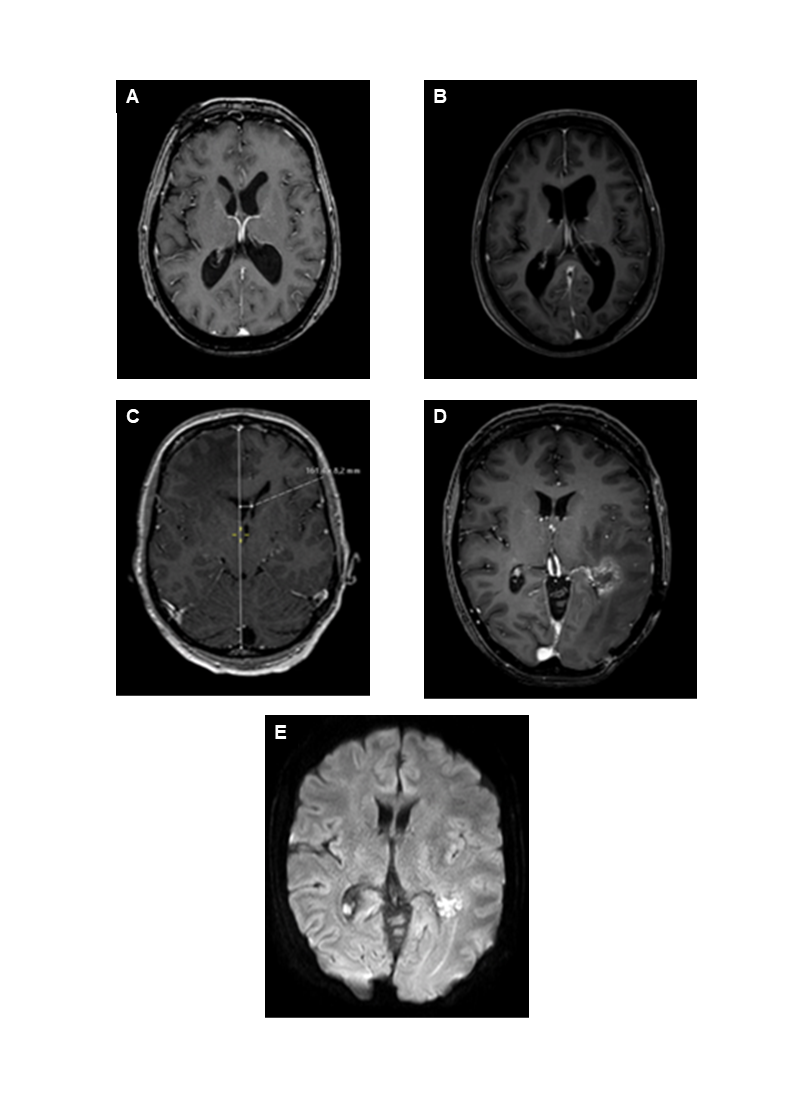
*

***The following examples of radiological determinants are shown****: MRI scan 6 months before progression (A), hydrocephalus compared to previous scan (panel A) (B), midline shift > 5 mm (C), gadolinium-enhancing tumor with non-enhancing center (D), and diffusion restriction in non-enhancing part (E).*
